# The antioxidant, wound healing properties and proteomic analysis of water extracts from the tropical cyanobacteria, Nostoc NIES-2111_MUM004

**DOI:** 10.1007/s13205-022-03448-0

**Published:** 2023-02-02

**Authors:** Su Chern Foo, Zi Sheng Lee, Michelle Khai Khun Yap, Ji Wei Tan

**Affiliations:** School of Science, Monash University Malaysia, Jalan Lagoon Selatan, 47500 Bandar Sunway, Selangor Malaysia

**Keywords:** Antioxidant activity, Biochemical profile, Cell culture study, Liquid chromatography mass spectroscopy, Water extracts, Wound healing

## Abstract

Cyanobacteria bioactive compounds are chemical treasure troves for product discovery and development. The wound healing effects and antioxidant capacities of water extracts from *Nostoc* NIES-2111_MUM004 were evaluated via in vitro wound scratch assay and three antioxidant assays respectively. Results showed that the water extracts were protein-rich and exhibited good antioxidant properties in ABTS radical scavenging (11.27 ± 0.205 mg TAE g^−1^ extract), Ferric reducing antioxidant power (1652.71 ± 110.71 mg TAE g^−1^ extract) and β-carotene bleaching assay (354.90 ± 31.80 mg TAE g^−1^ extract). Also, extracts were non-cytotoxic in concentrations up to 250 µg/mL as reflected in cytotoxicity assay. Importantly, water extracts showed considerable proliferation and migration activity at 125 µg/mL with wound closure rate as high as 42.67%. Statistical correlation revealed no significant relationship (*p* > 0.05) between protein fraction and the wound healing properties, confirming that phycobiliproteins were not solely responsible for wound healing activities. Subsequent Q-TOF-LCMS analysis identified six protein families involved in enhancing the proliferation and migration of epithelial cells. These findings are antecedent in the uncovering of continuous supplies of bioactive compounds from new and sustainable sources. Ultimately, enriching the microalgae menu for applications in pharmaceutical, nutraceutical and cosmeceuticals.

## Introduction

The growing health and well-being industry demands for alternative and sustainable bioactive resources. Cyanobacteria are ancient photosynthetic microorganisms that live in aquatic environments and have been touted as the next generation sustainable bioactive cell factories. For example, *Spirulina*-origin health products are household names due to their wide use in supplements, feed, and food. Microalgae have 10 × faster growth rate than terrestrial crops and does not compete with land to grow. This competitive advantage highlights their ability to address global challenges i.e., to sustain and nourish a growing human population. Cyanobacteria are called cell factories because they synthesize their own macronutrients (protein, carbohydrate, and lipids) and micronutrients (phycobiliproteins, beta-carotene, small bioactive molecules, etc.). Past studies reported that the cyanobacteria bioactive e.g., phycobiliproteins from *Spirulina platensis, Oscillatoria* sp*.* and *Nostoc commune* imparted antioxidant activities (Shanab et al. 2012; Carlos et al. 2021; Tseng et al. 2021). Whereas in wound healing, Neyrinck et al. (2017) showed that the protein fraction of S*pirulina platensis* facilitated macrophage activation while reducing inflammation in wound sites. Jeong et al. (2019) also reported that *Spirulina* sp. crude proteins promoted migration and proliferation in IEC-6 cells by activating the EGFR/MAPK signalling pathway.

With over 200,000 documented cyanobacteria species, less than a handful of cyanobacteria species are commercialized. *Spirulina* currently dominates as one of the most widely cultured cyanobacteria species (Silva et al. 2020). Whilst the pharmaceutical mechanism of action of *Spirulina* are researched to a certain extent, this leaves much research work to reveal the true biotechnological and pharmacological potential of other cyanobacteria species. In the meantime, it is crucial for us to safeguard the genetic diversity of under-discovered species which could have future uses in the changing climate. From a valorisation perspective, a diverse cyanobacteria source can diversify the microalgae menu to ensure continuous and steady supply of bioactive in the microalgae value chain for a circular bioeconomy. As such, one high potential but under-discovered cyanobacteria genus is *Nostoc. Nostoc* was traditionally eaten as a salad in several parts of Asia. Despite investigative efforts reporting the many applications of *Nostoc* bioactive, research studies on the wound healing properties by *Nostoc* genus remains under-represented. Further exacerbated by mixed bioactive leading to inconsistent study findings, this lends credence to the urgency for an exploratory study, starting with in vitro evaluation assays and supported by in-depth compound characterization work.

Liquid-chromatography-mass spectrometry (LC–MS) profiling characterizes bioactive compounds present in the post extraction process step. This analytical instrumentation technique is widely used in the pharmaceutical and food industry (Chen and Pramanik 2009). LCMS is important in the identification of compounds to aid in exploration of extracts action while lending an important perspective on the role of each compound in relation to bioactivity (Toyoshima et al. 2019). In LCMS proteomics, polar or water extracts are subjected to enzymatic cleavage where resulting products undergo post-digestion separation to produce a homogeneous mixture of peptides. Samples are then analysed using a mass spectrometer, enabling the identification of small organic molecules in proteins. This study employs the use LC–MS for the first time for our species of interest, and a crucial milestone for targeted protein profiling of the compounds responsible for wound healing.

The main scope of this study is to determine the wound healing properties and to identify the bioactive peptides (besides phycobiliproteins) present in the water extracts of *Nostoc* NIES 2111_MUM004. The sub-objectives include (1) to characterize the biochemical compositions followed by antioxidant capacity evaluation of the water extract and; (2) to identify the bioactive compounds responsible for wound healing properties in the water extract. As the first biochemical and bioactivity report for *Nostoc* NIES 2111_MUM004, this research will explore the least assessed cyanobacteria genus, where correlation studies will unveil the link between cyanobacteria peptides and wound healing properties.

## Materials and methods

### Materials

Trolox (ChemCruz, United States), 90 mm filter paper, 0.45 µm pore size (Advantec®, Malaysia), 75% ammonium sulphate (R&M, UK), Dimethyl sulfoxide purchase from Emsure®, 99.8% ethanol (Systerm®, Malaysia), potassium persulfate (Bendosen, Malaysia), TPTZ (Sigma Life Science, Germany), ferric chloride, FeCl_3_ (Systerm, Malaysia), sodium acetate (Systerm®, Malaysia), HCl (Friendemann®, United States), chloroform (Nacalai tesque®, Japan), Tween-20 (Systerm®,Malaysia), CCK assay (Sigma Aldrich, United states).

### Microalgae biomass preparation

*Nostoc* NIES 2111_MUM004 microalgae were provided by the Microalgae culture collection unit, School of Science, Monash University Malaysia. This species was isolated from Sunway South Quay Lake (Bandar Sunway, Selangor, Malaysia; 3^o^3′44.280″ N, 101^o^36′12.731″ E) where it was taxonomically identified in the lab. This was followed by molecular confirmation using 18S ribosomal DNA sequences with reference to method by Minhas et al. (2016) with slight modifications. The DNA extracted from the native microalgae cells were compared with published gene sequences from the NCBI database to determine nearest homologous sequences. *Nostoc* NIES-2111_MUM004 will be referenced as *Nostoc*_MUM004 in the remainder of this scientific article. Upon species confirmation, microalgae cells were cultivated in controlled conditions i.e., 25.15 ± 2.1 °C in standardized BG11 nutrient media (Stanier et al. 1971) at pH 6.5 ± 0.5 and illuminated by eight cool white 32 W fluorescent lamps at 47.31 µmol photons m^−2^ s^−1^ in 12:12 h (light/dark) photoperiod. Microalgae cultures were grown for 14 days and harvested by filtration using a filter cloth with 10 µm pore size. Harvested cells were placed in 50 mL falcon tubes and centrifuged at 10000 rpm for 5 min to remove excess water in the culture before freezing at − 80 °C. After 24 h, biomass was placed in a freeze dryer (Labconco, United Kingdom) for 3 to 4 days at 4 °C to obtain the freeze-dried biomass of *Nostoc*_MUM004. The biomass was stored at − 20 °C prior to analysis.

### Aqueous extraction of water extracts

One gram of freeze-dried biomass was added to 100 mL of Milli-Q grade water in a 250 mL conical flask. The flask was placed in an ultrasonic ice bath for 5 s, 30 kHz followed by 15 s resting time. This cycle was repeated for 30 min. After that, samples were left in an orbital shaker (Stuart, United States) for 24 h at room temperature. The next day, extracts were filtered through 90 mm filter paper, 0.45 µm pore size (Advantec®, Malaysia). Proteins from the filtrate were precipitated using 75% ammonium sulphate (R&M, UK). The protein precipitates were then dissolved in pre-chilled to 4 °C Milli-Q grade water and pooled prior to buffer exchange in a dialysis tubing for 2 h at room temperature. The dialysis buffer was changed and continued with another 2 h dialysis. The dialyzed sample was collected, and protein concentration was measured by Bradford assay (Harlow and Lane 2006), freeze dried and labelled as water extracts in subsequent experiments.

### Biochemical analysis

Freeze-dried biomass was measured to 0.05 mg using an analytical balance (Sartorius, Germany).

### Total protein content

Bradford method by Chen and Vaidyanathan (2013) was followed. In brief, 1 mL of methanol, 0.5 µL sodium hydroxide (1 N) and 20 µL phosphate buffer (0.05 M) were added to the weighted biomass and kept in an incubator at 100 °C, for 30 min. Then, samples were cooled down and 0.1 mL of the samples were collected for protein quantification. The blank was prepared by BSA powder (Vivantis Tech, Malaysia) at a concentration range of 20, 40, 60, 80, 100 µg/mL. One mL of Bradford reagent was added into 200 µL of samples and standards, respectively. Samples and standards were stored at room temperature for 10 min. The absorbance was recorded at 595 nm, then results expressed as percentage of total weight of cyanobacteria biomass.

### Total carbohydrate content

Carbohydrate quantification method by Chen and Vaidyanathan (2013) was followed. The standards were prepared at 40, 80, 120, 160 and 200 µgmL^−1^. To 0.2 mL of samples, 0.5 mL of 3,5-Dinitrosalicylic acid (DNSA) reagent was added to all treatments and standard. All samples were placed in a boiling bath for 15 min. Carbohydrate yields of biomass were assessed through a colorimetric method. The concentration of carbohydrate was determined through colour intensities of the samples at 540 nm. Besides, concentration was determined by standard curve as a reference. Standard curve was constructed through various concentrations of glucose. The results expressed the percentage of total weight of cyanobacteria biomass.

### Total lipid content

The modified method by Bligh and Dyer (Axelsson and Gentili 2014) was followed. Freeze-dried biomass (50 mg) were mixed with 10 mL of chloroform (Nacalai tesque, Japan) and 5 mL of methanol (J. Kollin Chemical, United Kingdom). The mixture was sonicated for 30 min. 4 mL of sodium chloride was added into the samples to make up the chloroform: methanol: sodium chloride at the ratio of 2: 1: 0.8. The upper chloroform layer was removed using 200 µL pipette and placed on a pre-weighted aluminium plate. Extracts were fully dried through a stream of nitrogen at room temperature. Samples were weighted, and lipids yield of samples were calculated. Total lipids of biomass calculated through weight difference before and after of extraction in grams. The results expressed the percentage of total weight of cyanobacteria biomass.

### Antioxidant capacities of *Nostoc*_MUM004 water extracts

#### ABTS antioxidant assay

The ABTS (2,2’-azino-bis-3-ethylbenzthiazoline-6-sulphonic acid) radical scavenging method by Foo et al. (2015a) was followed. Standard curve was prepared with Trolox (ChemCruz, United States) with concentration range from 3.125, 6.25, 12.5, 25, 50 and 100 ppm. 10000 ppm *Nostoc*_MUM004 water extracts were prepared by dissolving 10 mg of freeze-dried extract in ethanol. The ABTS radical was generated by adding 50 mL, 7 mM of ABTS stock solution with 2.45 mM potassium persulfate (Bendosen, Malaysia) then left in the dark for 24 h. The ABTS working solution with the reading of 0.7 ± 0.05 at 734 nm through dilution with ethanol. Twenty µL of samples and 200 µL added into the microplate and left in dark for reaction within 10 min. The intensity of radical-samples mixtures measured at absorbance of 734 nm. Antioxidant activity of aqueous and non-aqueous extracts expressed in mg Trolox Equivalent/gram dry weight (mg TEg DW^−1^).

#### Ferric reducing antioxidant power (FRAP) assay

The method referred to Foo et al. (2015b). Standard curve was prepared by Trolox with concentration at 3.125, 6.25, 12.5, 25, 50 and 100 ppm. The concentration of 1250 ppm and 625 ppm prepared through dilution with the solvent of each sample. The FRAP reagent was prepared with mixture of acetate buffer (pH 3.6), 10 mM TPTZ (Sigma Life Science) and 20 mM ferric chloride, FeCl_3_ (Systerm, Malaysia). 300 mM acetate buffer is formed by mixing 0.16 g of sodium acetate (Systerm®, Malaysia) into 100 mL of 0.28 M acetic acid. The pH of acetate buffer was regulated by hydrochloric acid (HCl) and sodium hydroxide (NaOH) to 3.2pH. TPTZ solutions were prepared by adding 0.31 g of TPTZ to 100 mL of 40 mM HCl (Friendemann®, United States). Ferric chloride stock solution prepared by adding 0.135 g of FeCl3 to 25 mL of distilled water. Fifty µmicroliters of samples mixed well with 150 µL of FRAP reagent. Then colour intensity of samples was quantified at 593 nm. The FRAP activity calculated and expressed in mg Trolox Equivalent/gram dry weight (mg TE/g DW).

#### β-carotene bleaching (BCB) assay

The method referred to Foo et al. (2017). Standard curve was prepared by Trolox with a concentration range from 3.125, 6.25, 12.5, 25, 50 and 100µgmL^−1^. Sample concentrations of 625 ppm and 31.25 ppm were prepared through dilution with the solvent of each sample. 6 mL of β-carotene solution formed by solubilizing 6 mg of β-carotene in 10 mL of chloroform (Nacalai tesque®, Japan). The solution was added into 120 mg linoleic acid to 1200 mg of Tween-20 (Systerm®, Malaysia). After that, 50 mL distilled water was added into dried mixture to form β-carotene-linoleic acid emulsion Solution dried with nitrogen gas to remove excess chloroform for 30 min. Next, 1 mL of emulsion was transferred into 1.5 mL centrifuge tube with 20 µL of samples and left for incubation by a water bath for 60 min at 50 °C. Samples colour intensity quantified spectrophotometrically at 470 nm. The antioxidant activity of extract/ fraction was expressed in mg Trolox Equivalent/gram dry weight (mg TE/g DW).

### HaCaT cell line culture

HaCaT cells were cultured in T25 cell culture flask containing complete DMEM media (supplemented with 15% FBS, 100 UmL^−1^ penicillin, and 100 µgmL^−1^ streptomycin), and was incubated in an incubator with the conditions of 37 °C, 5% carbon dioxide. The cell line underwent subculture every 3–5 days depending on the confluent of the cell (80–90%) by observing under an inverted microscope.

### Cell viability assay (CCK) assay

The purpose of CCK cell viability (Sigma Aldrich, United states) assay was to determine the non-cytotoxic concentration of extracts for wound scratch assay later. Firstly, HaCaT cell line was seeded in 96well plate with density of 1 × 10^6^ cell/mL, the cells were cultured together with Dulbecco’s Modified Eagle’s Medium (DMEM) media for 24 h. HaCaT cells were then treated with *Nostoc_*MUM004 water extracts at a concentration from 31.25, 62.5, 125, 250, 500 to 1000 µgmL^−1^. Treated cells were incubated for 72 h at 37 °C supplemented with 5% CO_2_. The cell viabilities were checked every 24 h by CCK-8 solution and quantified with a spectrophotometer at 570 nm (Pelin et al., 2018). Cell viability was expressed as percentage compared to control group using the following formula:

$${\text{Cell}}\;{\text{Viability}}\,(\% ) = \frac{{{\text{Corrected}}\;{\text{Absorbance}}\;{\text{of}}\;{\text{Samples}}/{\text{Treatment}}}}{{{\text{Corrected}}\;{\text{Absorbance}}\;{\text{of}}\;{\text{Negative}}\;{\text{control}}}} \times 100$$

### In vitro wound healing assay

The cell scratch assay is an in vitro method used to test the effect of bioactive compounds on proliferation and migration of epithelial cells (Liu et al. 2019). The assay was carried out following previous published protocol (Ritto et al. 2017) with slight modifications. HaCaT cells were seeded at 4 × 10^5^ cells/well in 24-well plates with incomplete medium (DMEM and penicillin/ streptomycin). After 24 h of incubation, a small linear scratch was drawn across the confluent monolayer by gentle scraping with a sterile p200 pipette tips (care was taken during the scratching process to ensure uniform size and distance for all samples). Cells were rinsed with PBS to remove cellular debris before adding the media with *Nostoc* sp. fluid and aqueous extract at the concentration of 31.25, 62.50 and 125.00 µgmL^−1^. A 5% of FBS was used as a positive control group and cells without treatment were considered as the negative control. The vehicle control was prepared by mixing incomplete media with sterile distilled water. All treatment groups were incubated for 24 h at 37 °C, 5% of carbon dioxide. After 24 h, images of migrated cells were taken using a digital camera connected to inverted microscope to observe the closure of wound area. Images were captured at 24, 48 and 72 h and analysed using ImageJ software (Scion Corporation, United Stated). The percentage of wound healing closure rate was calculated through migration and proliferation of HaCaT cells using the following formula:

$${\text{Wound}}\;{\text{healing}}\,(\% ) = \frac{{{\text{Corrected}}\;{\text{Absorbance}}\;{\text{of}}\;{\text{Samples}}/{\text{Treatment}}}}{{{\text{Corrected}}\;{\text{Absorbance}}\;{\text{of}}\;{\text{Negative}}\;{\text{control}}}} \times 100$$

### LC–MS analysis of Nostoc water extracts

The chromatography analysis began where a reaction mixture was prepared by mixing 15 µL of 50 mM ammonium bicarbonate with 1.5 µL of 100 mM dithiothreitol (DTT). Then 10 µL of protein samples were added into mixture. Next, 0.5 µL of UltraPure water was added to make up the total volume of 27 µL. Then, the sample was incubated at 95 °C for 5 min. After that, extracts were cooled down at room temperature. Then, 3 µL of 100 mM iodoacetamide (IAA) was added into mixture and incubated in dark at room temperature for 20 min. One µL of MS sequencing grade trypsin (0.1 µgµL^−1^) was added into samples and incubated for 37 °C for 3 h. Another 1 µL of trypsin was added and underwent protein digestion overnight at 30 °C. The digested peptides were then cleaned up by ZipTip® Pipette tips (Merck Millipore) with reverse pipetting technique, according to manufacturer’s protocol. The cleaned peptides were subjected to Agilent 1200 HPLC-Chip/MS Interface coupled with Agilent 6550 iFunnel Q-TOF LC/MS. Approximate 1 µL of peptide sample was loaded into Agilent Large Capacity Chip, 300A, C18, 160nL enrichment column (75 µm x 150 µm) analytical column. The flow rate was 4 µL/ min. The MS parameters such as ion polarity was positive and capillary voltage (Vcap) was 1900 V. Fragmentor voltage set for analysis was 360 V where gas temperature was 325 °C, the drying gas flow was 5.0 Lmin^−1^. The mass spectra generated were searched against Uniprot *Nostoc*_MUM004 on Swissprot + TrEMBL.

### Statistical analysis

The research was performed in triplicates. Data were expressed as mean ± standard deviation. One-way ANOVA and Tukey post hoc tests were used to indicate significant differences at *p* < 0.05 and *p* < 0.001. Pearson correlation was used to reveal possible relationships between biochemical, antioxidant and wound healing capacities. GraphPad Prism 8 was used to plot the graphs. Image J was used to measure the total area of HaCaT cell proliferation and migration.

## Results

### Biochemical profile and antioxidative properties of investigated water extracts

**Fig. 1:**
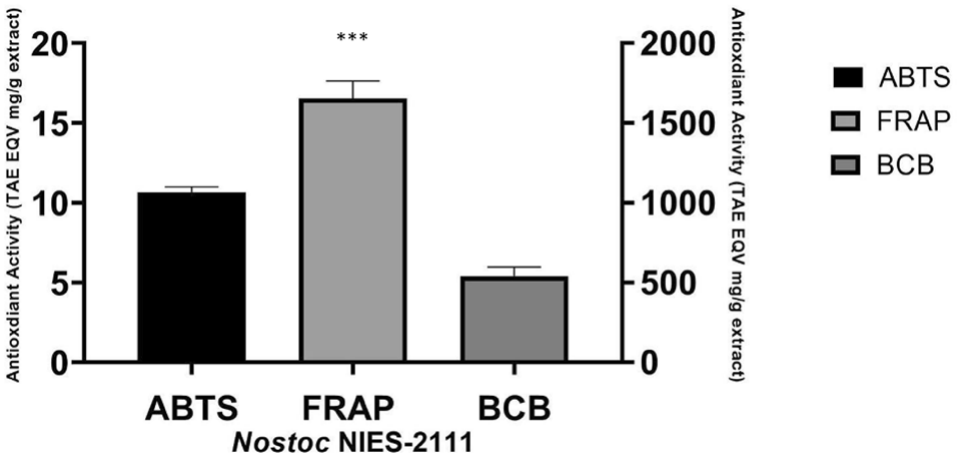
ABTS radical scavenging activities, ferric reducing activities, and β-carotene bleaching antioxidant activities from *Nostoc*_MUM004 water extracts. ***indicate significant differences at *p* < 0.05 confidence level

Biochemical composition of *Nostoc*_MUM004 for protein, carbohydrate and lipids were reported to be 73.938 ± 2.32%, 16.333 ± 1.72% and 2.662 ± 0.71%. The protein levels were found to be higher than previous studies reporting *Nostoc commune* (31.90%) (Jerez-Martel et al. 2017). Figure 1 shows antioxidant activities of the water extracts in *Nostoc*_MUM004. The primary antioxidant ABTS assay showed that water extracts from the investigated *Nostoc* sp. had good radical scavenging activities (10.65 ± 0.34 mg TAEg^−1^ extract). This radical scavenging activity was comparable to that reported by Shanab et al. (2012) in *Nostoc muscorum*. What was interesting however was, the FRAP activity (1652.71 ± 110.71 mg TAEg^−1^ extract) was approximately 8 times higher than past reports in *Nostoc muscorum* (220.26 mg TAEg^−1^ extract) (Hajimahmoodi et al. 2010). Further to radical scavenging and reducing ability, the water extracts were also found to impart good protective abilities. This was evidenced in the secondary antioxidant assay i.e., β-carotene bleaching assay where, water extracts at 537.98 ± 58.15 mg TAEg^−1^ extract were able to slow down β-carotene discoloration in the water-linoleic acid emulsion system.

### Water extracts showed high cell viability and wound healing properties at 250 µg/mL

**Fig. 2:**
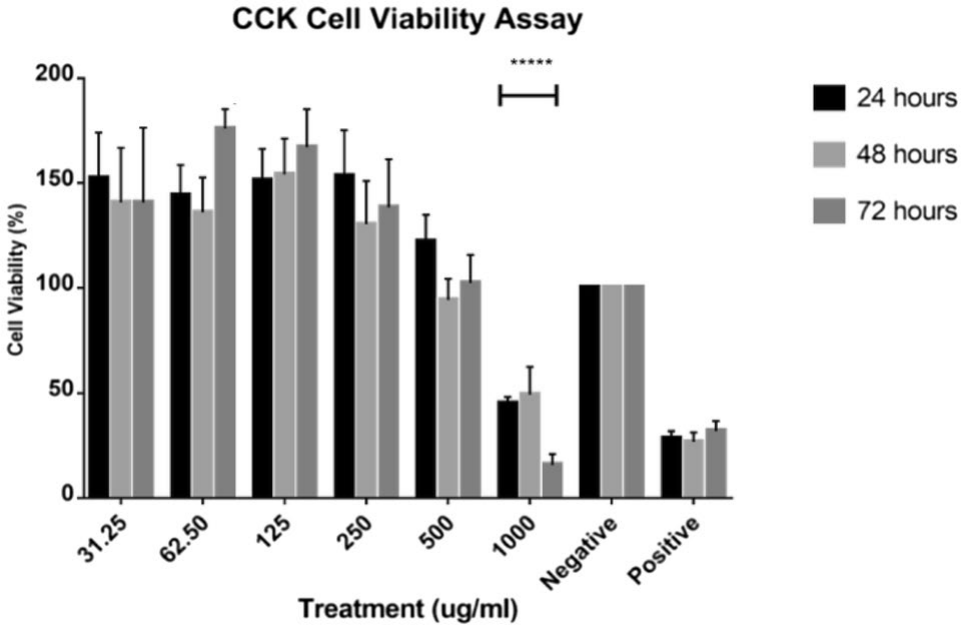
*Nostoc*_MUM004 water extracts treatment on HaCaT cell line on 24 h, 48 h and 72 h at an increasing concentration of 31.25, 62.50, 125, 250, 500 and 1000 µgmL^−1^. The results were expressed as mean of cell viability percentage (%) ± SD. *****p < 0.05 compared to the negative control group

**Fig. 3:**
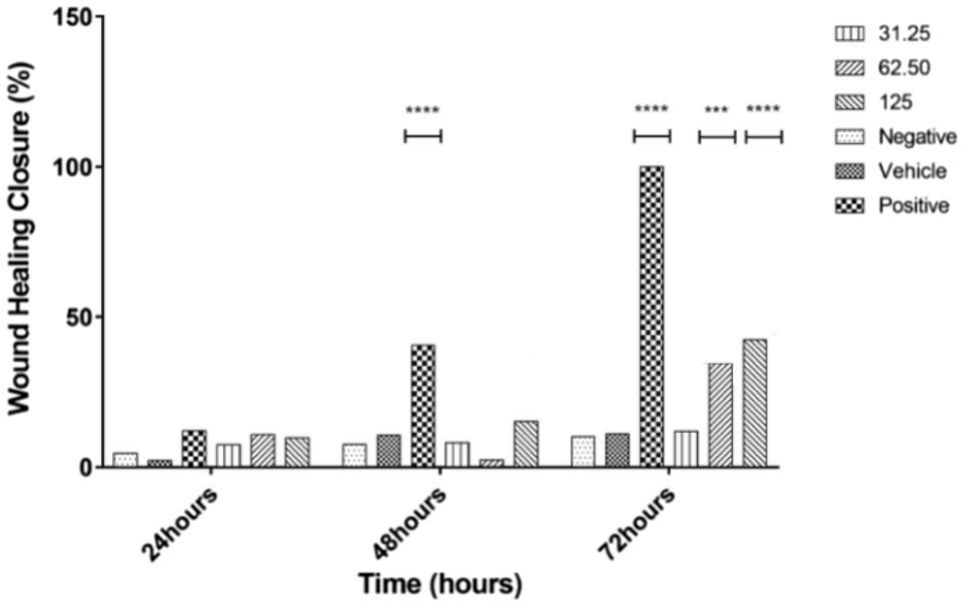
Water extracts of *Nostoc*_MUM004 (31.25, 62.50 and 125 µgmL^−1^) treated on HaCaT cell in wound scratch assay at 24 h, 48 h and 72 h. Wound healing closure percentage (%) assessed through area of cell proliferation and migration. The results were expressed as a mean of wound healing closure percentage ± SD. ***p < 0.05 and ****p < 0.001 compared to the negative control group

Prior to scratch assay, the CCK-8 cytotoxicity assay was conducted to determine the non-toxic concentration range for *Nostoc*_MUM004 water extract. Figure 2 showed significant decline (*p* < 0.05) in cell viability up to 250 µgmL^−1^ at 24 h, 48 h and 72 h. However, at higher treatment concentrations (500 µgmL^−1^ and 1000 µg mL^−1^), the cell viability started to decline more steeply. For example, at 1000 µg/mL treatment concentration and relative to the negative control group, cells showed significantly lower (*p* < 0.05) cell viability for all three time points. From here, the water extracts at 31.25, 62.5 and 125 µg mL^−1^ were selected for the subsequent in vitro wound scratch assay. The wound healing effects from selected concentrations were quantitatively evaluated by percentage of area in which HaCaT cells migrated and proliferated (Fig. 3). This was complemented by representative morphological images of HaCaT cells at selected timepoints (Table 1). At 31.25 mL^−1^, the percentage wound closure did not show a significant (*p* > 0.05) wound healing effect at 24 h, 48 h or 72 h. At 62.50 µg mL^−1^, the percentage wound closure only showed significant effect (*p* < 0.05) relative to the negative control group at 72 h. However, at 125 µg mL^−1^, percentage wound closure started showing significant effect (*p* > 0.05) as early as 24 h i.e., from 11.14 to 21.32%. Subsequently, the wound closure percentage peaked at 42.67% at 72 h and was significantly higher (*p* < 0.05) than other the two lower treatment concentrations. From here, it was clear that the 125 µg mL^−1^ treatment group exhibited the best wound healing effect by promoting highest proliferation and migration in HaCaT cells.

**Table 1:** Wound closure (yellow outline) exerted by *Nostoc*_MUM004 water extracts in HaCaT cell scratch assay over time

	0 h	24 h	48 h	72 h
Positive				
Negative				
Vehicle				
125 µg/mL				
62.5 µg/mL				
31.25 µg/mL				

Scale bar in all representative figures is 20 µm

### LC–MS Chromatography analysis

Table 2 shows the assignment of peptide sequences to the respective protein family in the water extracts of *Nostoc*_MUM004. Overall, the LCMS analysis demonstrated a total of 28 protein families belonging to *Nostoc* sp, where 6 protein families were identified to contribute to the wound healing properties (Table 3).

**Table 2:** Assignment of tryptic digested peptides of *Nostoc*_MUM004 to protein families by Q-TOF-LC–MS/MS accessed on 13 March 2020 using Uniprot *Nostoc* NIES-2111, Swissprot + TrEMBL

Protein name	Accession number	Peptide sequence	m/z	RT	Query cover (%)	*E* value
AAA family ATPase [*Nostoc sp*. NIES-2111]	WP_096680967.1	ETLQFSK	426.7574	21.26	100	2.7
DUF4114 domain-containing protein [*Nostoc* sp. NIES-2111]	WP_096683138.1	IESSTSK	376.244	20.48	100	4.5
ferredoxin-NADP + oxidoreductase [*Desmonostoc* sp. PCC 7906]	BAG69182.1	AVRQLQK	421.7616	17.07	71	0.23
sensor histidine kinase [*Nostoc* sp. NIES-2111]	WP_096679804.1	LNTIHSK	271.53	17.07	100	9.9
insulinase family protein [*Nostoc* sp. PA-18-2419]	WP_138499051.1	ALQAEIK	332.9	19.93	100	1.6
CHAT domain-containing protein [*Nostoc* sp. NIES-2111]	WP_096681907.1	SLSLLGGTVV	473.346	43.71	100	0.012
nitrate reductase [*Nostoc linckia*]	WP_099069194.1	FHTPDGRAR	352.9	28.22	100	0.003
family 10 glycosylhydrolase [*Nostoc piscinale*]	WP_062289469.1	ESAIARNGK	473.346	43.71	100	0.028
MULTISPECIES: 16S rRNA (cytosine(967)-C(5))-methyltransferase [unclassified *Nostoc*	WP_067768222.1	QYIRSDEASP	292.1898	20.5	100	4.00E-04
serine/threonine protein kinase [*Nostoc* sp. NIES-2111]	WP_096678107.1	KNIVWLLILALL	353.0538	45.88	100	2.00E-06
GAF domain-containing protein [*Nostoc* sp. NIES-2111]	WP_067770071.1	LPSLLGLHFPAD	427.2707	21.2	100	6.00E-09
type I-D CRISPR-associated helicase Cas3’ [*Nostoc* sp. NIES-2111]	WP_096680030.1	HAGFSAYQAFV	599.2462	30.46	100	6.00E-05
PAS domain S-box protein [*Nostoc* sp. NIES-2111]	BAY37603.1	LAELELLQQK	296.945	21.24	100	1.00E-03
type I restriction-modification system endonuclease [*Nostoc cycadae*]	WP_103124733.1	QDAGIEVGR	236.9427	23.05	100	2.20E-02
MULTISPECIES: HAMP domain-containing protein [unclassified *Nostoc*]	WP_067768065.1	FTSAGGK	223.1751	21.51	100	4.70E + 00
type I restriction-modification system endonuclease *[Nostoc cycadae*]	WP_103124733.1	DFNDYVSK	494.3077	22.92	100	2.20E-02
30S ribosomal protein S4 [*Nostoc* sp. PA-18-2419]	WP_138504283.1	AYPPGQHGQNR	306.848	29.06	100	2.00E-05
ABC transporter ATP-binding protein [*Nostoc* sp. NIES-3756]	WP_067768587.1	YEVWELIRQLK	370.0337	48.66	100	3.00E-06
DUF3854 domain-containing protein [*Nostoc* sp. NIES-2111]	WP_096682741.1	IAQNIYSK	234.9618	12.49	100	4.40E-02
SDR family NAD(P)-dependent oxidoreductase [*Nostoc* sp. NIES-2111]	WP_096681600.1	ATLAAIAATGSK	537.8521	43.99	100	3.00E-04
alkanesulfonate monooxygenase [*Nostoc* sp. NIES-2111]	BAY36072.1	ELQSSKTVER	294.864	19.25	100	1.00E-03
polynucleotide adenylyltransferase region [*Nostoc linckia* NIES-25]	BAY77126.1	TPALQTR	262.8481	17.43	100	1.20E + 00
M1 family metallopeptidase [*Nostoc* sp. NIES-2111]	WP_096681268.1	LSAIRALGK	232.9722	2.76	100	5.50E-02
NAD(P)/FAD-dependent oxidoreductase [*Nostoc* sp. ‘Lobaria pulmonaria (5183) cyanobiont’]	WP_104906479.1	VNPAQLR	200.1826	31.6	100	8.20E-02
ATP-dependent DNA helicase RecG [*Nostoc* sp. ‘Peltigera malacea cyanobiont’ DB3992]	WP_099100676.1	DLLFYYPR	543.9307	48.59	100	5.00E-03
hypothetical protein [*Nostoc* sp. NIES-2111]	WP_096678986.1	HFGTPIR	414.1471	28.88	100	2.90E-01
ParB/RepB/Spo0J family partition protein [*Nostoc* sp. NIES-2111]	WP_096683715.1	TASSTSNSESNNYK	373.1037	47.28	100	3.00E-07
hypothetical protein [*Nostoc* sp. NIES-2111]	WP_096678378.1	TATAECWGR	263.9357	17.77	100	2.00E-03
hypothetical protein [*Nostoc* sp. NIES-2111]	WP_096683493.1	DIGCYGIVFR	300.8999	36.14	100	9.00E-05

**Table 3:** Protein families identified from Q-TOF-LC–MS chromatography and their functions to wound healing

Protein	Function	References
AAA-ATPase	Play critical roles in rearrangement leading to proper migration of cells. Enhance proliferation of keratinocytes cells	Khong et al. (2020)
Ferredoxin-NADP + oxidoreductase	Transfer electrons from photosystem I to NADPH during photosynthesis	Mosebach et al. (2017)
Sensor histidine kinase	Catalyse phosphorylation of histidine residue to detect signals such as change in environment to alter the states of cell	Jaubert et al. (2017)
CHAT domain containing protein	Control reactive oxygen homeostasis and regulation of redox reaction and oxidative stress	Méheust et al. (2016)
HAMP domain-containing protein	Play a role in oxidative stress regulation with histidine kinase	Randhawa and Mondal (2013)
GAF domain-containing protein	Structural module responsible for binding allosteric regulatory molecules Regulating proliferation, migration, inflammatory response, and cytoskeletal rearrangements	Pardoux et al. (2021)
Nitrate reductase	Improved wound healing on diabetic subcutaneous tissue	Schäffer et al. (1997)
Family 10 glycohydrolase	Protect DNA of smooth muscle cells in the vascular system from oxidative stress	Sharifi et al. (2013)
Serine/threonine protein kinase	Catalyst serine and threonine to serine phosphate and threonine phosphate. Redox modification by oxidant and antioxidant	Heng (2011) Rang et al. (2016)
Type 1-D CRISPR associated helicase Cas3’	Interaction selectivity and non-covalent bond with compound between and within the cells Interacting selectivity non-covalent bond with any nucleic acid	Elsherbini and Ezzat (2020)
Type 1 restriction-modification system endonuclease	Immune system against foreign DNA, bacteriophages, involvement in processes including DNA replication and repair	Morgan et al. (2008)
ABC transporter ATP-binding protein	Transport system to maintain cell viability via regulating osmotic strength in the cell, responsible for both suprabasal differentiation and in p63-expression keratinocytes migrating	Huls et al. (2009)
SDR family NAD(P) dependent oxidoreductase	Catalyst of redox reaction, revert chemical reaction, oxidation states of atoms	Vidal et al. (2018)
M1 family metallopeptidase	Play a vital role in wound healing. Promote human keratinocyte migration on fibroblast collagen	Salo et al. (1994) Matoori et al. (2021)
ParB/RepB/Spo0J family partition protein	Interact selectivity and non-covalently with DNA	Socea et al. (2021)

## Discussion

### High protein content and Ferric reducing capacities in *Nostoc*_MUM004

The high protein yields (72.94%) from our investigated species suggested that culture conditions used in this study favoured protein synthesis in the cells. This was ideal as the macronutrient of focus in this study was protein. Another reason for the higher yield in protein in this study when compared to Jerez-Martel et al. (2017) reporting 34% protein yield in *Nostoc commune,* could also be attributed to the method of protein extraction. The sonication step used in our study was shown to be effective in disrupting the cyanobacterial cell wall subsequently releasing the contents into the water solvent as compared to vortexing used by past studies. For the antioxidant assays, water extracts were able to exert good primary and secondary antioxidant activities by scavenging radicals, transferring electrons to reduce Fe^2+^ to Fe^3+^ as well as, act as sacrificial antioxidants to confer protection in an emulsion system that mimics phospholipids in the cell. This study also observed exceptionally high FRAP activities Indeed, the growth phase of the microalgae biomass from which the bioactive are extracted from can have significant effects on antioxidant activities. For example, Badr et al. (2019) reported *Nostoc* sp. harvested on the 16th day had the highest levels of caffeic, ferulic and gallic acids. Although phenolic profiling was outside the scope of this study, a major reason accounting to the higher FRAP activity was due to growth phase of when the microalgae biomass was collected. In this study *Nostoc*_MUM004 was harvested on the 14th day of culture for high protein content as revealed in our previous work (Lim et al. 2022).

### Water extracts exert wound healing effects on HaCaT cell line

The investigated *Nostoc*_MUM004 exhibited comparable wound healing properties to that of its commercial counterpart, *Spirulina platensis*. For example, Liu et al. (2019) reported that crude protein extract of *Spirulina platensis* elicited a wound closure of 45% whereas this study reported wound closure of 42.67% at 125 µg mL^−1^. Moreover, Jeong et al. (2019) found that crude protein extract of *Spirulina platensis* promoted cell migration at 12.5–50 µg mL^−1^ concentration range. Nevertheless, it is worthy to exercise caution when comparing with past work as results can differ even within the same species due to experimental differences in type of animal cells, passage numbers and its’ culturing conditions, cell seeding concentrations, all which can introduce confounding variability to the results.

### Pearson relationship between biochemical, antioxidant and wound healing properties

The relationship between wound healing to antioxidant activities by cyanobacteria extracts has been evidenced in studies e.g., Demay et al. (2021). During acute or chronic injuries, cells in the wound microenvironment generate reactive oxygen species (ROS) to protect against foreign organisms from wound infection. Redox is a regulative mechanism to manage oxidative stress and optimise the wound environment to enhance healing (Dunnill et al. 2017). Thus, a balanced level of oxidants and antioxidants are crucial in the wound healing physiological process (Xu et al. 2020). While oxidant acts as messengers in the wound healing pathway, excessive oxidants can destroy wound structure and biocidal reactive oxygen species generated in the wound microenvironment during inflammation retards the wound healing process (Kapuścik et al. 2013). To balance this, microalgae bioactive compounds contain antioxidant properties useful to reduce inflammation risk. In doing so, can avoid extensive tissue damage and promote wound healing.

To better understand this relationship, Pearson correlation were used to statistically show the relationship between biochemical, antioxidant and wound healing. This study found there were no significant correlation (*p* > 0.05) between biochemical to antioxidants or antioxidants to wound healing. This was contradictory to past work reporting biochemical compounds to be responsible for its antioxidant activities (Guerreiro et al. 2020; Jerez-Martel et al. 2017) or positive correlation between antioxidant activities and wound healing effects (Parwani et al. 2014; Gunes et al. 2017). From here, we postulate from the findings of this study that the lack of correlation between antioxidants to wound healing could be due to the experimental design where post aqueous extraction, would selectively exclude phycobiliproteins using a 0.2 µm pore size polyethersulfone (PES) filter. This was based on the fact that phycobiliproteins sizes are in the range of 300 kDa (Balti et al. 2021) thus would remain on the PES filter. Filtrates were collected for subsequent testing using wound healing assay. This step was deliberate to reduce phycobiliproteins interference to the results and to test our hypothesis whether *Nostoc* small proteins alone were responsible for the wound healing effects. Despite the lack of relationship between antioxidants to wound healing, the same statistical test revealed that proteins from water extract showed a significant correlation (*p* < 0.05) to wound healing properties. This confirmed our hypothesis that compounds responsible for wound healing properties imparted by *Nostoc*_MUM004 was more than phycobiliproteins. With this new lead, a targeted LCMS proteomic characterization study was conducted for the investigated water extract.

### Protein families in Nostoc_MUM004 related to wound healing pathway

Table 3 lists the protein families identified in *Nostoc*_MUM004 water extract and associates each to their role in wound healing. A previous study by Khong et al. (2020) showed AAA-ATPase played an integral part of the physiological process in wound healing by accelerating the cell motility in turn promoting epithelial cell migration. The same study found that absence of AAA-ATPase impaired the wound healing effect of cells shown in the wound scratch assay. Also, the ABC protein can impart anti-inflammation by suppressing macrophages that damages tissue structure (Huls et al. 2009). On the other hand, the serine/threonine protein kinase is a stress response enzyme that is involved in wound healing by redox modification (Heng 2011). Serine/threonine protein kinase also plays key physiological roles in cell growth, proliferation, apoptosis, and cell motility (Rang et al. 2016) and therefore influences the wound healing process. In addition, it is noteworthy that M1 metallopeptidase protein family was found in the water extracts of *Nostoc*_MUM004. Previous studies revealed that the M1 metallopeptidase enzyme plays a key role in wound re-epithelialization by regulating extracellular matrix degradation and deposition (Salo et al. 1994). Also, M1 family metallopeptidase activates NF-kB, mitogen-activated protein kinase and focal adhesion kinase pathways to shorten wound closure rate while regulating chronic and acute inflammation (Matoori et al. 2021; Saqib et al. 2018; Chatzizacharias et al. 2007).

Taken together, findings revealed that the water extracts of *Nostoc*_MUM004 showed exceptionally high antioxidant activity e.g., ferric reducing activity at 1652.71 mg TAE g^−1^. This could be partly attributed to the presence of antioxidant enzymes in the extracts as revealed in LCMS. For example, ferredoxin NADP + Oxidoreductase and SDR family NAD(P)-dependent oxidoreductase enzymes are involved in the intracellular electron transports (Wang et al. 2010), and are also potent cytoprotective agents to prevent the epithelial cells from oxidation damage. This was attainable through the delay of damage on tissues and cells caused by ROS, possibly by electron transfer conferred by the water extracts as reflected from the FRAP assay.

Our previous findings showed how fucoxanthin from diatoms activated the anti-proliferative pathway in HepG2 liver cancer cells by targeting multiple cellular pathways (Foo et al. 2019). Similarly for wound healing, the water extract from *Nostoc_*MUM004 could impart a characteristic ROS-regulating pathway in wound healing. Thus, it would be interesting to follow through the findings with Enzyme Linked Immunosorbent Assay (ELISA) and western blotting starting with interleukins, key cell signalling molecules in tissue regeneration (Arshad et al. 2020). Besides that, inserting an *in-silico* digestion step e.g., SpirPep discovery workflow before wet lab enzyme digestion could prove useful. This *in-silico* step was demonstrated by Anekthanakul et al. (2019) where angiotensin I converting enzyme (ACE)-inhibitory peptides were computationally cleaved from the amino acid sequences of *Spirulina platensis*. In doing so, systematically reduced cost for lab consumables and shortening the bioactive peptides discovery process during the screening phase.

## Conclusions

The investigated *Nostoc*_MUM004 water extracts are rich in proteins and exhibited a dual role as both primary and secondary antioxidants. Extract concentrations within the range of 31.25–125 µgmL^−1^ did not result in cytotoxicity. Importantly, the wound scratch assay revealed wound healing effects by water extracts increased at a concentration dependent manner i.e., 31.25 µgmL^−1^ > 62.5 µgmL^−1^ > 125 µgmL^−1^. Therefore, the recommended concentration of water extracts to exhibit best wound healing effect at a non-toxic concentration to cells is 125 µgmL^−1^. Further proteomic profiling using LC–MS revealed bioactive proteins responsible for the revealed wound healing properties. Indeed, the presence of six protein families related to wound healing were confirmed i.e., AAA-ATPase (responsible for cell migration), serine-threonine protein kinase (cell growth, proliferation, and motility), ABC-transporter protein (promotes faster healing rate), M1 metallopeptidase (role in wound re epithelialization) and both ferredoxin NADP + Oxidoreductase and SDR family NAD(P)-dependent oxidoreductase (cytoprotective agents). This preliminary but important work will be antecedent to further studies where RT-PCR and ELISA assays are warranted to elucidate the wound healing pathway exerted by *Nostoc* proteins. Overall, it is envisioned that the study findings will facilitate and advance the diversification of the cyanobacterial menu for commercialization as nutraceuticals, cosmeceuticals, and pharmaceuticals. This will ultimately expand the market potential for a microalgae-based biorefinery that promotes circular bioeconomy.

## Data Availability

The data that support the findings of this study are available on request.

## Declarations

### Research involves human or animals participation

This research work did not involve human participation and/or animals.
